# Merkel Cell Polyomavirus (MCPyV) and Its Possible Role in Head and Neck Cancers

**DOI:** 10.3390/biomedicines13051180

**Published:** 2025-05-12

**Authors:** Sara Passerini, Sara Messina, Ugo Moens, Valeria Pietropaolo

**Affiliations:** 1 Department of Public Health and Infectious Diseases, “Sapienza” University of Rome, 00185 Rome, Italy; messina.2140775@studenti.uniroma1.it; 2 Department of Medical Biology, Faculty of Health Sciences, University of Tromsø, UiT-The Arctic University of Norway, 9037 Tromsø, Norway; ugo.moens58@gmail.com

**Keywords:** head and neck cancer, oral squamous cell carcinoma, oncogenic viruses, Merkel cell polyomavirus, Merkel cell carcinoma, non-MCC tumors

## Abstract

Despite significant progress in its prevention, diagnosis, and treatment, head and neck cancer (HNC) remains a major global health issue due to its multifactorial pathogenesis. Indeed, HNCs have been found to be associated with different environmental and lifestyle factors, as well as with infection with oncogenic viruses. To date, seven viruses are recognized for their tumorigenic properties and have been proposed as implicated in HNC development, including Merkel Cell Polyomavirus (MCPyV). MCPyV is well recognized as the major etiological agent of Merkel cell carcinoma (MCC), a rare but rapidly metastasizing skin neoplasm. Specifically, in almost 80% of MCC cases, viral genome integration occurs, and a truncated form of Large T Antigen (tLT) is expressed. Although MCC is a rare cancer, MCPyV is a ubiquitous virus, widely distributed among the human population. Therefore, a plausible role of the virus has been proposed, even for other tumors. The current review provides an overview of the available data describing the presence of MCPyV in non-MCC tumors, such as HNCs, with the aim of elucidating the potential contribution of MCPyV to oral cancer. Understanding the role of viral infections in the etiology of cancer opens up the opportunity for developing preventive measures and targeted therapies that effectively address HNC progression while reducing treatment-related side effects.

## 1. Introduction

Head and neck cancers (HNCs) are the seventh most common type of cancer in the world, representing 5% of cancers worldwide. HNCs encompass a broad range of malignancies arising from the upper aero-digestive region, including the oral cavity, pharynx, larynx, nasal cavity, paranasal sinuses, and salivary glands [1,2]. Thyroid cancer, skin cancers affecting the head and neck (HN), and ear cancers are anatomically located in this region, and they are typically managed separately from traditional HNCs. Moreover, lymphomas, while not conventionally classified as HNCs, may also affect the HN region [3]. There are many types of cancers concerning HN, which are classified based on their anatomical location using the International Classification of HN tumors (5th edition) from the World Health Organization (WHO) [4]. Oral cancer (OC) represents the most frequently occurring malignant tumor in the HN region and the sixth most common cancer with high mortality in the world [5]. Moreover, nearly 90% of all HNCs are squamous cell carcinomas (SCC) [2,6]. Oral squamous cell carcinoma (OSCC) is an epigenetic and genetic disease representing the most prevalent form of OC [7]. The mouth and tongue are the most common sites where OSCC typically develops [8,9]. The clinical management of HNCs is highly challenging due to their multifactorial etiology, heterogeneous nature, and varied treatment responses [10]. Despite improvements in diagnostic techniques and treatments, HNCs continue to be a global health burden, with more than 660,000 new cases and 325,000 deaths annually [4,11,12], highlighting the need for a deeper understanding of their underlying mechanisms. The burden of HNCs varies significantly across geographical locations, generally linked with the confounding risk factors associated [13]. For example, the incidence and mortality rates of oral cavity and laryngeal cancers are higher in Europe compared to the United States [13]. Clinical and epidemiological studies have identified a broad spectrum of risk factors associated with HNCs, with approximately 85% of cases attributed to cigarette smoking or the use of other tobacco products, such as cigars, pipes, and smokeless tobacco [14,15,16]. Additionally, alcohol consumption increases the risk for the development of HNCs by 10-fold [17]. Epidemiological studies indicate that consuming tobacco and alcohol may have a combined effect in causing HNCs [18]. Recently, the incidence of OSCC has been rising among young male patients with less exposure to tobacco and alcohol, emphasizing the role of other risk factors. Indeed, diets with low fiber, malnutrition, poor oral hygiene, and genetic disposition have been associated with the development of HNCs [19,20].

Among the various factors involved in HNC development, oncogenic viruses have emerged as significant contributors, playing a pivotal role in both the initiation and progression of these malignancies [9]. Seven oncogenic viruses are currently recognized, including the following five DNA viruses: human papillomavirus (HPV), Epstein–Barr virus (EBV), Kaposi’s sarcoma herpesvirus or human herpesvirus 8 (KSHV or HHV-8), Merkel cell polyomavirus (MCPyV), and hepatitis B virus (HBV) [9]. HPV is predominantly associated with cancer in the female and male genitals, but it was also linked to oral and oropharyngeal cancer [21,22]. EBV is the etiological factor of Burkitt’s lymphoma and nasopharyngeal cancer, whereas KSHV is the cause of Kaposi’s sarcoma [23,24]. Hepatitis B infection is associated with the development of hepatocellular carcinoma [25]. The role of MCPyV in cancer is discussed in Section 2. Moreover, there are two RNA oncoviruses, hepatitis C virus (HCV) and human T-lymphotropic virus type 1 (HTLV-1) [26,27]. Chronic HCV causes inflammation, which may ultimately lead to liver cancer [28]. HTLV-1 is associated with T-cell malignancies such as adult T-cell leukemia/lymphoma and HTLV-1-associated myelopathy/tropical spastic paraparesis, with marginal direct contribution in HNCs [29]. Oncoviruses induce cellular transformation by several mechanisms, including disrupting cell cycle regulation, evading immune surveillance, and promoting genomic instability [30]. In addition, human immunodeficiency virus (HIV), although not considered an oncovirus, destroys the immune system, leading to increased risk of cancer development, called HIV-associated cancers [31].

Different subtypes of HNCs display unique viral profiles and molecular signatures, reflecting their different biological behaviors and treatment responses [32]. Understanding the interaction between oncogenic viruses and host factors is crucial for creating targeted therapeutic approaches that successfully address HNC progression while minimizing treatment-related side effects. Improvements in detection techniques, such as highly sensitive polymerase chain reaction (PCR) assays, next-generation sequencing (NGS) [33], and advanced imaging [34], have revolutionized the isolation and characterization of viral involvement in HNCs, providing prompt and more accurate diagnosis, more accurate prognostication, and the development of personalized treatment approaches, thereby ultimately improving patient outcomes.

The current review will focus on the available data describing the presence of MCPyV in non-MCC tumors such as HNCs, aiming to provide a comprehensive overview of the potential contribution of MCPyV to OC.

## 2. Merkel Cell Polyomavirus (MCPyV) and Merkel Cell Carcinoma (MCC)

Polyomaviruses (PyVs) are a family of non-enveloped viruses with a circular double-stranded DNA genome of approximately 5000 base-pairs. The name derives from the observation that the first isolated member of this family was able to induce multiple (*poly*) tumors (*oma*) when injected into animal models [35]. More than 10 different human polyomaviruses (HPyVs) have been isolated so far, and although the oncogenic potentials of some of them in cell cultures and animal models have been reported, MCPyV is the only PyV directly associated with a human neoplasm [36,37].

First described in 2008, MCPyV (or human polyomavirus 5) is a small naked DNA virus recognized as the major causative agent of Merkel Cell Carcinoma (MCC), a rare but aggressive neuroendocrine skin cancer [38]. Indeed, about 80% of MCC cases are virus-induced cases, whereas a lower percentage of tumors do not harbor MCPyV DNA and/or proteins and are caused by UV-induced tumorigenic point mutations [39]. MCPyV’s genome encompasses two coding regions with a Non-Coding Control Region (NCCR) located between them [40]. The early region drives the expression of Large T (LT) and small T (sT) antigens. LT is a phosphoprotein that is absolutely required for the replication of the viral genome and is involved in the transcriptional regulation of the viral genes [41,42]. sT plays an auxiliary role for LT and can interfere with LT’s function by inhibiting protein phosphatase 2A (PP2A), thereby affecting the phosphorylation of LT [43]. Two additional proteins, 57 kT antigen and the Alternative LT Open reading frame (ALTO), are encoded by the early region, but their exact functions remain unclear [44]. The late region encodes for the capsid proteins, Viral Protein 1 and 2 (VP1 and VP2), and for two mature microRNAs, MCV-miR-M1-5p and MCV-miR-M1-3p, and it is able to downregulate early gene expression [45]. MCPyV miRNAs are expressed at low levels in virus-positive MCC tumors, and their contribution to MCPyV-mediated oncogenesis is still unclear [46]. Interposed between the two coding regions, there is the NCCR which comprises the origin of viral DNA replication, the promoter and enhancer elements for transcriptional regulation of viral genes during infection [47]. While rearrangements in the NCCR of other HPyVs such as BKPyV (or human polyomavirus 1) and JCPyV (human polyomavirus 2) have an impact on the transcriptional activity of the promoter, thus affecting the virulence of the virus and resulting in pathogenic viral strains, limited data are available about the plausible correlation between NCCR mutations and MCPyV-associated diseases [48].

MCPyV primary infection usually occurs in early childhood, either by the respiratory tract or through direct skin contact, and it can persist throughout adult life. MCPyV infection is innocuous in most people [49]. MCPyV infection is widespread, as serological studies reported that 50–80% of the healthy adult population has antibodies against the virus [50,51,52,53]. The virus is continuously shed from the skin, and cell culture studies have demonstrated that the virus can replicate in dermal fibroblasts [54,55]. In non-tumor conditions, MCPyV infects host cells by maintaining its genome in an episomal form, and low copy numbers of non-integrated MCPyV DNA have been detected in many healthy tissues, such as the adrenal gland, spleen, bone marrow, stomach, gallbladder, pancreas, heart, and aorta, although the authentic cell tropism of MCPyV is unknown [37,56,57].

As previously mentioned, MCPyV was first isolated from Merkel cell carcinoma (MCC) by the group of *Chang and Moore* [38]. They found that in all their tumor samples, the viral DNA was clonally integrated into the cancer cell genome, showing that integration was an early event during oncogenesis since it must have occurred before the expansion of the tumor cells [38,58]. Viral integration occurs in random sites by accidental genome fragmentation during viral replication and leads to mutational events in the MCPyV genome, which prompt carcinogenesis [39,59,60]. Subsequent studies by several groups revealed that 80% of all examined MCC samples contained integrated MCPyV [44,61]. The tumors expressed LT and sT and are considered key viral oncogenes in MCPyV-driven tumorigenesis [47]. However, whereas a wild-type sT was expressed, a C-terminal truncated form of LT (tLT) was produced due to nonsense or frameshift mutations generating premature stop codons [38]. Like other HPyVs, MCPyV tLT preserves the N-terminal J domain and the LXCXE motif and therefore the ability to bind and inhibit pRb. tLT also possesses an origin-binding domain, a nuclear localization signal (NLS), and a helicase binding domain but lacks the p53-binding region [62]. Consequently, integration disrupts the late region so that no infectious particles are generated in MCCs, whereas LT truncation eliminates the replicative function but maintains the oncogenic properties [44,63]. Indeed, tLT deregulates the cell cycle and apoptosis, thereby leading to the proliferation of tumor cells [63]. Specifically, pRb binding is recognized as essential in MCPyV-mediated oncogenesis [64]. Normally, pRb regulates the G1/S cell cycle transition by sequestering the E2F transcription factor. MCPyV LT binding to pRb leads to an inappropriate activation of E2F and uncontrolled cellular proliferation [40]. Experimental studies involving LT knockdown reported the inhibition of the growth of MCC cells, which cannot be rescued by the expression of a mutant LT protein unable to bind pRb, supporting the crucial role of LT-pRb interaction in tumorigenesis [65]. In vitro studies showed that neither full-length nor tLT was capable of inducing cellular transformation. The C-terminal region causes DNA damage and stimulates the host DNA damage response; therefore, it contains anti-tumorigenic properties. This may explain why this region is removed in virus-associated tumors [66].

Several observations prove that sT plays a pivotal role in MCPyV-driven oncogenesis. Indeed, the knockdown of MCPyV sT expression in MCC cell lines abrogated cell proliferation, and sT alone was sufficient to transform rodent fibroblasts and induce tumor formation in transgenic mice [67,68]. sT comprises a protein phosphatase 2A (PP2A) binding domain that promotes the modulation of the cell cycle [69]. Independent of the PP2A domain, studies showed that sT induces the hyperphosphorylation of eIF4E-binding protein 1 (4E-BP1), deregulating cap-dependent translation [ 68]. sT also interacts with MYCL and the EP400 histone acetylase and chromatin remodeling complex, thus affecting gene expression in MCC cells [70]. Moreover, a unique domain of sT, recognized as the LT stabilization domain, confers the capability to interact with a series of tumor suppressors such as FBW7, β-TrCP, and CDC20, thus leading to enhanced viral replication and oncogene activation [71]. Finally, sT can inhibit the NFκB pathway by interfering with the regulatory subunit of PP4 and stimulate cell mobility and invasiveness [72,73,74]. Taken together, in vitro and animal studies, together with the detection of wild-type sT in virus-positive MCC, suggest that sT plays the predominant role in the oncogenic process of MCC, whereas LT is required to maintain the tumor cell growth [44].

## 3. Merkel Cell Polyomavirus in Non-Cancerous Tissues and in Non-MCC Tumors

The high seropositivity in the human population [51,52,53]; the fact that MCPyV represents a part of the human skin microbiome [54]; the widespread prevalence of the virus, although with a relatively low viral load, in various non-cancerous tissues of the body [57]; and its role in the development of MCC prompted researchers to investigate the possible role and presence of MCPyV in non-MCC cancers.

The earliest observation connecting MCPyV with non-MCC was the isolation of MCPyV DNA in non-melanoma skin cancers from immunosuppressed patients [75]. Specifically, MCPyV DNA and transcripts have been isolated at varying levels in many non-melanoma skin cancers, including squamous cell carcinomas [75,76,77,78,79,80,81] and basal cell carcinomas [75,82,83,84]. The presence of the virus was further reported in rare cases of kerato-acanthoma [78,82,85], Kaposi’s sarcoma [86,87], poro-carcinoma [79,88], atypical fibro-xanthoma [82], and Langerhans cell sarcoma [89]. In contrast, melanomas are not related at all with MCPyV [90,91,92].

As predicted, due to its capability of replicating in cultures of lung fibroblasts [55], MCPyV has been detected in tumors of the respiratory system. Among them, non-small-cell lung carcinoma was more closely associated with the presence of the virus [93,94,95], despite the fact that none of the analyzed specimens display any LT protein expression [96], except for the detection of tLT in two non-small-cell lung carcinomas [93]. Interestingly, one of these two samples exhibited a unique duality, containing both episomal and integrated MCPyV DNA, while expressing both the full-length and tLT protein [93]. MCPyV was rarely reported in lymphatic system cancer, except tonsillar squamous cell carcinoma (32%; *n* = 150) [97,98] and thymoma (15.2%; *n* = 46) [93,99,100]. Tumors of the circulatory system were further found to harbor MCPyV sequences; more specifically, one acute myeloid leukemia specimen was tested positive for MCPyV DNA [101]. However, other studies reported no traces of the virus in this type of tumor [102]. In addition, MCPyV transcripts were detected in chronic lymphocytic leukemia cells [103,104,105,106], whereas six samples expressed tLT mRNA and two of them also harbored the full-length form of LT [103]. Despite limited data being available about MCPyV prevalence among reproductive system-related tumors, some studies reported MCPyV DNA in prostate cancer [107,108], breast cancer [109], and cervical cancer [110]. In contrast, when investigating tumoral tissues from the digestive tract, MCPyV sequences were found with a slightly higher frequency, as in the case of esophagus cancer (45.1%) [111], liver cancer (62%) [107], or salivary gland cancer (26.2%) [112]. MCPyV was detected with a low prevalence in the bladder (4%; *n* = 147) [107,113,114] and renal cancer (3.7%; *n* = 81) [107,114], with lower levels of viral loads in tumor cells when compared to MCCs. Furthermore, MCPyV has sporadically been detected in tumors of the skeletal system, such as Ewing sarcomas, chordomas, chondrosarcomas, and rhabdosarcomas [115,116]. In the context of soft tissue-related tumors, MCPyV has been investigated only within a limited case of desmoplastic tumors; however, no traces of viral DNA have been detected in this type of cancer [115]. Moreover, when exploring MCPyV prevalence in tumors of the nervous system, viral transcripts were detectable in a few schwannomas, meningiomas, glioblastomas [117], and neurofibromas [118], while no association to the virus was recognized in neuroblastomas [113,115,119].

## 4. Merkel Cell Polyomavirus in Non-MCC Tumors Associated with HNCs

To date, several studies have tried to clarify the role of HPyVs in the pathogenesis of HNCs. More specifically, several groups have focused on MCPyV, which is the only HPyV clearly associated with a human neoplasm [38]. However, MCPyV prevalence and plausible contribution to HNC development have not been fully established and are currently under investigation.

In a pilot study by Hamiter et al., MCPyV DNA was found in 28.6% of patients with SCC of the tongue (*n* = 6/21), whereas all biopsies taken from the normal base of tongue in non-cancerous subjects were negative for viral DNA [120]. In another study, the presence of MCPyV and other HPyVs, including HPyV6 and HPyV7, was evaluated in non-malignant tonsils (*n* = 108) and tonsillar SCC (*n* = 112). The results showed an increased prevalence of MCPyV DNA in cancer specimens compared to non-malignant tissues (35.7% vs. 10.2%), while the prevalence of the other two HPyVs was identical in tumor and non-cancerous tissues [97]. Herberhold *and colleagues* evaluated the DNA presence and load of HPyVs in tonsillar SCC (*n* = 38) and non-cancerous tonsillar tissue (*n* = 40). MCPyV and HPyV6 were identified in a higher ratio in cancer versus non-malignant tissue; however, the differences were non-significant [98]. Afterwards, a pilot study from Iran evaluated 50 HNSCCs and reported MCPyV DNA in 16% of cases, with a higher viral load in stage III tumors, suggesting that viral infection may affect only a subset of HNCs and thus highlighting the need for additional investigations [121]. Another study, by Munoz et al., assessed 120 HNSCCs samples from the Chilean population and reported MCPyV in 12.5% of the patients [122]. BKPyV and JCPyV were rarely detected, with only one positive case of BKPyV and none of JCPyV. Notably, MCPyV was almost absent in oral brushes from individuals without cancer, proposing a possible correlation between MCPyV and HNC development [122]. Poluschkin et al. [123] studied the prevalence and viral DNA loads of simian virus 40 (SV40 or *betapolyomavirus macacae*), JCPyV and BKPyV by quantitative PCR (qPCR), and all HPyVs with a novel Multiplex method in 82 HNCs samples with known HPV status and disease-specific survival (DSS). JCPyV was found to be the most prevalent PyV, present in 37% of HNSCC, and the most common regions were the lips (80%), larynx (67%), and oral cavity (59%). BKPyV was found only in one case with low viral DNA copies. SV40 was isolated from 60% of lip specimens and 20.7% of specimen with low viral loads. Overall, 86% of JCPyV-positive samples were co-infected with HPV, with no impact on DSS. The multiplex method identified additional HPyVs in five samples as follows: four HNCs samples were positive for MCPyV and one sample for HPyV6. The carcinomas were located in the lips, maxillary sinus, and tongue. In contrast to the studies described above, a recent study by Mulder et al. did not report MCPyV in HNCs patients (*n* = 119) without smoking and/or drinking history by immunohistochemistry (IHC) analysis for LT expression, whereas HPV and EBV were isolated from oropharyngeal and nasopharyngeal SCCs, respectively [124]. Likewise, Windon et al. investigated the prevalence of MCPyV in oral cavity cancers (OCC) (*n* = 126). Among the 44 cases examined for LT expression by IHC, none of the tumors expressed MCPyV, thus rejecting MCPyV as a causative agent for the development of OCC [125]. Hasani Estalkhi et al. [126] carried out a study in Northern Iran, exploring MCPyV in 114 oral cavity biopsies, including cancerous (OSCC, dysplasia) and non-cancerous lesions [oral lichen planus (OLP), and oral irritation fibroma (OIF)]. They reported MCPyV DNA in 20% of OSCCs, 21.4% of dysplasia samples, 24.1% of OLP samples, and 30.6% of OIF samples, indicating a plausible involvement of MCPyV in both cancerous and non-cancerous oral lesions. These findings suggest the plausible contribution of MCPyV in oral cavity pathogenesis, warranting further investigation. Finally, in 2024, Passerini et al. [127], to improve the understanding of MCPyV in oral cavities, performed the detection and analysis of MCPyV DNA, transcripts, and miRNA on OSCCs and oral potentially malignant disorders (OPMDs). More specifically, for the first time, viral integration and LT truncation, two hallmarks of virus-mediated oncogenesis, were investigated in these types of tumors. The results showed MCPyV replication in both OSCC and OPMD, indicating the oral cavity as a site of replicative MCPyV infection, thus emphasizing the active role of this virus in the occurrence of oral lesions. Results obtained from all the above cited studies are summarized in Table 1 (additional data is available in Table S1).

## 5. Conclusions

In conclusion, the role of MCPyV in the induction of HNCs remains controversial. Despite the detection of viral DNA, transcripts, and proteins in malignant tissues, the contribution of MCPyV in tumors other than MCC is still a topic of debate, since the prevalence of the virus varies from study to study due to the different detection methods used. Moreover, due to the ubiquitous nature of the virus, MCPyV has also been isolated from non-malignant tissue. Consequently, a causative role for MCPyV in HNCs remains to be defined.

Indeed, so far, there is no gold standard test for the detection of MCPyV, and even the combined use of PCR and immunohistochemistry (IHC) shows suboptimal results. Previous research employing the two combined methods for detecting the virus in MCC tissues reported a higher positivity by PCR, supporting this technique is more sensitive and accurate. However, other studies observed a superior viral detection by IHC. These discrepancies may be attributed to the great diversity in PCR methodologies, including the use of primers targeting different viral sequences and the number of primer sets and variations in the cut-offs used to consider a test as positive [128].

The contribution of MCPyV in HN tumors other than MCC remains unclear for various reasons as follows: (a) the viral genome copy number is very low; (b) LT transcripts and protein are rarely reported; (c) tLT and viral genome integration, two hallmarks for virus-positive MCCs, have not been investigated in the majority of studies; (d) not all groups examining the same type of tumor could reproduce similar results about viral prevalence, load, or LT expression; (e) differences in results due to the use of fresh tumor tissues or formalin-fixed paraffin-embedded samples; (f) healthy adjacent non-tumor tissue was rarely examined; and (g) DNA extracted from tumor tissues may contain DNA from infiltrating non-tumorous cells such as T-cells, macrophages, and cancer-associated fibroblasts, potentially resulting in false positive PCR results.

**Figure 1:**
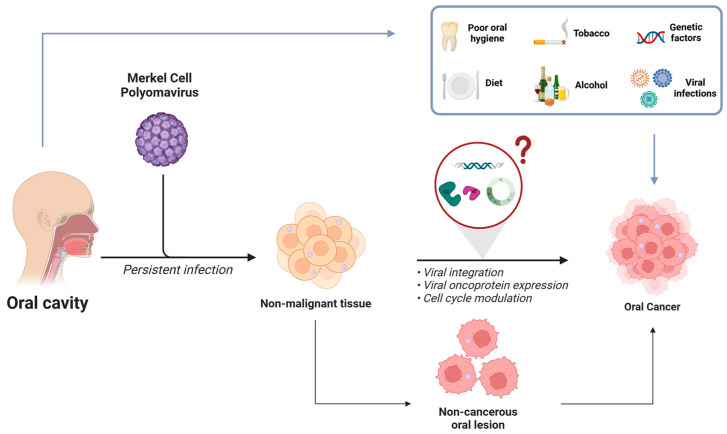
Illustration displaying the plausible role of MCPyV in oral pathogenesis, in conjunction with other risk factors (lifestyle, genetic factors, and viral infections). The figure reports the potential contribution of the virus to oral lesions and the hypothetical mechanism involved in virus-mediated oncogenesis in the HN region, including viral integration, viral oncogenic protein expression, and cell cycle modulation. Created in BioRender https://BioRender.com/ythycyq (accessed on 31 March 2025).

The available literature suggests that further meticulous studies, involving more samples, standardized protocols focused on MCPyV detection, viral integration analysis, and investigations of tLT ans sT expression, as well as comparisons to matching non-cancerous tissues, are needed to delineate the viral etiopathogenesis in the development of HNCs. A plausible role for MCPyV in oral carcinogenesis, in conjunction with other risk factors, including oncogenic viruses such as HPV and EBV or within the context of HIV infection, should be considered (Figure 1).

## Figures and Tables

**Table 1:** Prevalence of MCPyV in the HN region.

Tissue (*n*)	Prevalence *n* (%)	Sample Type	Viral Load (Copies/Cell)	Viral Integration	Method	References
Oral tongue SCC (21)	6 (28.6)	FFPE	NA	NA	PCR (LT)	[120]
Squamous cell tonsillar carcinoma (97)	33 (34)	FFPE	NA	NA	qPCR (LT)	[97]
Non-malignant tonsillar tissue (103)	10 (9.7)					
Non- malignant tonsillar tissue (40)	4 (10)	Biopsy	0.000004	NA	PCR (LT)	[98]
Tonsillar carcinoma (38)	8 (21.1)		0.000064			
SCC (50)	8 (16)	FFPE	0.0048	NA	PCR (LT, VP1), qPCR (LT), RT-PCR (LT)	[121]
Non-cancerous adjacent normal tissue (50)	1 (2)		0.000026			
SCC (119)	0 (0)	FFPE	NA	NA	IHC (LT)	[124]
Oral brushes (54)	1 (1.8)	FFPE	NA	NA	qPCR (LT)	[122]
SCC (120)	15 (12.5)					
OCC (126)	0 (0)	FFPE	NA	NA	IHC (LT)	[125]
OC (114) including	28 (24.6)	FFPE		NA	qPCR (LT)	[126]
SCC (35)	7 (20)		0.0232			
LP (29)	7 (24.1)		0.000272			
Dysplasia (14)	3 (21.4)		0.0202			
IF (36)	11 (30.6)		0.000257			
SCC (11)	3 (27.3)	FFPE	1.17 × 10^2^ *	No integration	qPCR (sT)	[127]
PMD (12)	4 (33.3)		1.74 × 10^2^ *			

* Viral load expressed as copies/mL. NA: not assessed.

## Data Availability

The data are contained within the article.

## Supplementary Materials

The following supporting information can be downloaded at: https://www.mdpi.com/article/10.3390/biomedicines13051180/s1, Table S1: Additional data. References [97,98,120,121,122,124,125,126,127] are cited in Supplementary Materials.
